# A review with updated perspectives on in vitro and in vivo wound healing models

**DOI:** 10.55730/1300-0152.2659

**Published:** 2023-08-10

**Authors:** Murni Nazira SARIAN, Nabilah ZULKEFLI, Mohamad Shazeli CHE ZAIN, Sandra MANIAM, Sharida FAKURAZI

**Affiliations:** 1 Institute of Systems Biology (INBIOSIS), Universiti Kebangsaan Malaysia (National University of Malaysia), 43600, Bandar Baru Bangi, Selangor, Malaysia; 2 Bioresource Technology Division, School of Industrial Technology, Universiti Sains Malaysia (Science University of Malaysia), 11800, Pulau Pinang, Malaysia; 3 Faculty of Medicine and Health Sciences, Universiti Putra Malaysia (University of Putra Malaysia), Serdang 43400, Selangor, Malaysia

**Keywords:** Wound healing, in vitro, in vivo, healing agent, therapeutics

## Abstract

A skin wound or perforation triggers a series of homeostatic reactions to safeguard internal organs from invasion by pathogens or other substances that could damage body tissues. An injury may occasionally heal quickly, leading to the closure of the skin’s structure. Healing from chronic wounds takes a long time. Although many treatment options are available to manage wound healing, an unmet therapy need remains because of the complexity of the processes and the other factors involved. It is crucial to conduct consistent research on novel therapeutic approaches to find an effective healing agent. Therefore, this work aims to cover various in vitro and in vivo methodologies that could be utilised to examine wound recovery. Before deciding on the optimal course of action, several techniques’ benefits, drawbacks, and factors need to be reviewed.

## 1. Introduction

A wound develops when the skin is punctured, which damages the body’s tissue. An injury results from a collapse of the skin’s protective function, which leads to an acute or a chronic wound. Chronic wounds impose complications on the healing process, whereas acute wounds can move through the typical sequential stages of wound healing. Chronic wound healing is hampered and becomes imprisoned, typically at the inflammatory phase, especially in hyperglycaemic conditions. The healing process for the wound fails to progress and is delayed. Several pathological elements, such as chronic illnesses like diabetes mellitus, are caused by impaired blood circulation and wound bacterial infection; these contribute to the persistent nature of chronic wounds (Wilkinson and Hardman, 2020).The stages of wound healing involve complex overlapping biological phases, namely haemostasis, inflammation, angiogenesis, and skin tissue maturation (Wallace et al., 2022). Haemostasis is an immediate response at the wound site to stop blood loss as soon as skin damage occurs. Vasoconstriction and the stoppage of further bleeding are components of the haemostasis stage. Following an injury, blood can escape the body through a cut on the skin. A temporary fibrin matrix is created to stop future bleeding. The tear is sealed in the blood vessel wall; platelets, cytokines, and other clotting components are produced and concentrated at the wound site (Wallace et al., 2022).

The inflammatory phase is part of the second stage of wound healing. Removing debris and preventing infection trigger a subsequent inflammation, which starts with the neutrophil influx and is aided by the mast cell release of histamine (Wilkinson and Hardman, 2020). To avoid infection, the wound site is cleaned of bacteria and possibly contagious microorganisms at this stage. In the initial stages of inflammation, the large number of neutrophils released and circulated at the wound site can prevent infection. Inflammation-related cell debris is removed by neutrophils. These contain granules of cells emitting powerful hydrolytic and proteolytic enzymes that can consume bacteria and foreign objects. Concurrently, inflammatory mediators, such as monocytes (which later develop into macrophages) will be drawn to the wound damage site to phagocytose germs and dead tissues. Keratinocytes migrate to cover the wound gap during the proliferative phase, blood vessels regenerate through angiogenesis, and fibroblasts replace the first fibrin clot with granulation tissue (Alhajj and Goyal, 2021; Dipietro, 2016; Landén et al., 2016). At this stage of recovery, regulatory T cells (Tregs) and macrophages are also essential. Ultimately, myofibroblasts produce total wound contraction, blood vessels retreat, and fibroblasts further remodel the matrix that has been formed. Through the production of endothelial cells, fibroblasts, growth factors, and keratinocytes, which multiply to close the wound area, the wound prepares to regenerate (angiogenesis) the lost tissue. Granulation tissue encourages collagen synthesis and introduces new scaffolding into the damage, which causes the wound margin to close and scar tissue to develop (Alhajj and Goyal, 2021).

**Figure 1:**
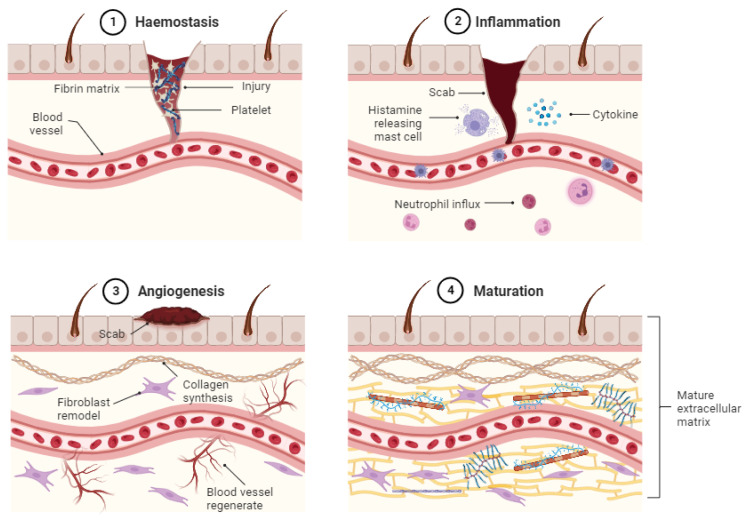
Wound healing phases: (1) haemostasis, (2) inflammation, (3) angiogenesis, (4) maturation.

The extracellular matrix (ECM) develops, matures, and gains mechanical strength during healing. The proliferation phase may be prolonged if the process is affected by interference during this phase. Depending on the size of the wound, the procedure known as maturation or remodelling, which is the last stage, may take weeks or months. The skin’s tensile strength increases throughout development, while the collagen remodels, multiplies, and matures. On the other hand, an excessive wound resulting in a disproportionate maturation stage produces keloids and hypertrophic scars (Wang et al., 2020). The wound healing phases are shown in Figure 1.

Meanwhile, the local macrophages in chronic nonhealing wounds, like diabetic foot ulcers, continue to have proinflammatory properties and remain in the inflammatory phase of the damage for an extended period. When their levels are consistently high, chemokines, interleukins, and cytokines cause tissue injury to deteriorate and extend the inflammatory healing stage. Skin openings facilitate microbial contamination and consequent local illness. A prolonged onset of the damage and a lack of prompt intervention delay the wound healing process. Prolonged wounds cause imbalanced amounts of growth factors and proteases, which are covered by a polysaccharide matrix, called a biofilm, that is secreted by bacteria. Three common bacteria—*Staphylococcus aureus* (*S. aureus*), *Pseudomonas aeruginosa* (*P. aeruginosa*), and haemolytic streptococci—play a significant role in prolonging chronic wounds. The biofilm may explain the failure of the use of antibiotics to treat chronic wounds. Persistent wound injuries require special attention and therapy to help them enter the proliferative phase.

## 2. Use of in vitro and in vivo models in wound healing

The ethical standards of scientific research forbid the use of humans as direct clinical subjects, especially those with a reduced wound healing capacity, when doing so could result in the subject being injured (Varkey, 2021). Simultaneously, the need for medication discovery, as well as research into pharmacological actions and characteristics, toxicological relevance, and drug efficacy, must be clinically demonstrated, particularly with regard to enhancing wound care management. As a result, using a model is a specific way of acquiring and comprehending the complexities of the wound healing process. At the same time, the efficacy of the wound-healing candidate must be established. Thus, several in vitro and in vivo models have been developed to examine the wound healing process. These models are crucial tools for enabling scientists to complete translational research with notable clinical studies that would improve wound care and management.

Before a drug product is commercialised or used for medical purposes, a step-by-step study is typically carried out, beginning with in vitro testing, in addition to preclinical and clinical evaluations. For example, fibroblasts, macrophages, keratinocytes, and endothelial cells can all be purchased commercially, such as from American Type Culture Collection (ATCC) (Stamm et al., 2016). Other researchers have employed in vitro models to create organ cultures (Al-Lamki et al., 2017). In contrast, in vivo models primarily use animal models (such as pigs, rabbits, rats, mice, or even zebrafish) to explore how wounds heal in tissues and organs (Grada et al., 2018), while clinical studies use humans as the study subjects (Food and Drug Administration, 2014).

The complexity of the wound healing process requires the adoption of both in vitro and in vivo models, despite their dissimilarities. In vitro wound healing models are regarded as rapid, simple, less costly, and entailing minimal ethical considerations (Stamm et al., 2016). This type of model allows a direct examination of how an environmental change or substance has affected the tissue without influencing any other tissue components (Ud-Din and Bayat, 2017; Shrivastav et al., 2018). This advantage means in vitro wound models are often applied to study a compound’s mechanism of action (Sorg et al., 2017). Previous researchers have studied the cell proliferation and migration activities of 3T3 fibroblast cells upon being treated with flavonoid glycosides, namely orientin, isoorientin, vitexin, and isovitexin, using an in vitro scratch assay (Che Zain et al., 2020). Some pharmacological agents or factors can also be investigated at different concentrations simultaneously using this type of model (Guo and Dipietro, 2010; Albrecht, 2020). Despite these advantages, in vitro models limit the evaluation of the dynamic wound healing process, such as haemostasis, inflammation, angiogenesis, and maturation. Although these models allow absolute control over environmental factors such as the temperature, artificial wound size, and treatment concentration, they cannot reproduce biological conditions such as enzymes, cells, and tissue interactions. Hence, these models are regarded as not clinically translatable as they make it difficult to deduce the results of wounded tissue in humans.

The limitations of in vitro models make in vivo models superior in terms of assessing wound healing potential. In vivo models are regarded as highly medically relevant if the wound is generated to mimic the wound seen in clinical practice. The wound healing processes closely resemble the same process seen in humans. For instance, skin wounds can be made to represent those found in human subjects. In vivo models allow the simultaneous examination of multiple wound healing factors in a complex environment. Unlike in vitro models, in vivo models are difficult to use in direct examinations of single-tissue components. The involvement of animal subjects requires strict procedures to obtain ethics approval from an Animal Ethics Committee. In the case of human skin wounds, these models only allow the creation of small, clean wounds.

This section discusses in vitro and in vivo models, concentrating on the benefits and disadvantages that have received the most attention. The section examines the variables to be considered, the benefits and drawbacks, the concepts to be adopted, and potential directions to be adopted in such investigations. Since each model replicates a particular stage of human wound healing, a study’s objectives must be considered when selecting appropriate in vitro and in vivo models (Cialdai et al., 2020). Other practical aspects, such as the financial resources available, the execution period, the space and physical facilities, the technical know-how, and the procurement, may aid in choosing the best models (Tottoli et al., 2020). Therefore, a researcher must be able to select the most pertinent model that accurately represents the wound conditions found in humans under the particular study parameters. The results from the most practical model may serve as preliminary proof or a leading indicator of any factor influencing the wound healing process. However, the flaws in each type of model mean that any established model can only be used to make general predictions. No model can accurately replicate a clinical scenario (Masson-Meyers et al., 2020).

## 3. In vitro models

**Figure 2:**
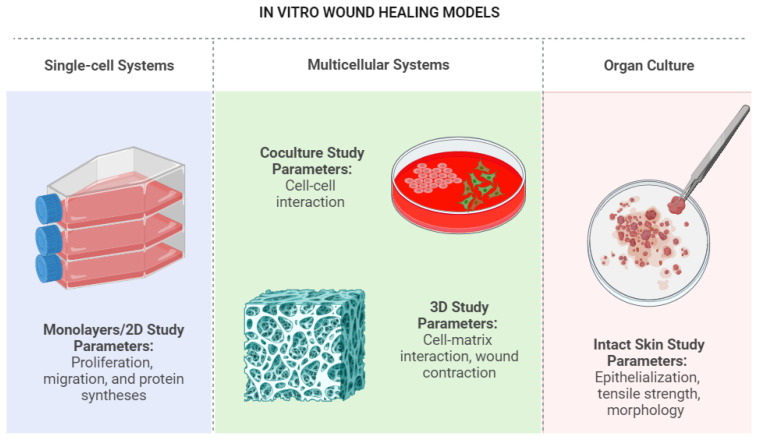
Summary of in vitro models used in wound healing studies.

The first rapid screening model for studying wound healing uses a single-cell system since this is quick, reliable, and affordable. The most frequently used technique is a scratch test assay, which enables the evaluation of wound closure and cell migration (a scratch is formed with a pipette tip on a confluent monolayer of cells). A wound healing concept or mechanism involving one or two cell types in response to one or more stimuli can be understood due to the development of in vitro models. The versatility of in vitro models enables studies of proliferation, migration, protein syntheses, cell-cell interaction, cell-matrix interaction, wound contraction, epithelisation, tensile strength, and morphology during wound healing. Studies have been conducted to quickly identify any potential wound healing effects of the chosen compounds or items using three main in vitro models, including single-cell systems (Cui et al., 2021), multicellular systems (Miranda-Azpiazu et al., 2018; Xiao et al., 2019), and organ cultures (Varani, 2012). These investigations can serve as a foundation for developing the models into additional in vitro models for studying wound healing. Figure 2 lists many of the in vitro models and wound representations used in wound healing studies.

### 3.1. Single systems: monolayers/2D wound healing assays

The essential aim of every 2D wound healing assay is to purposefully disrupt or destroy a confluent cell monolayer, leaving a cell-free area that cells can use to bridge and repair. Research into wound healing using 2D assays involves three fundamental principles: describing the harm sustained, observing the healing process, and data analysis (Stamm et al., 2016). Before wounding, the chosen monolayer cells are grown in confluence, indicated by cells covering 80%–90% of the dish surface, directly on the plastic surface of culture dishes with rich media. Confluent cells can be purposefully destroyed by making an artificial wound region that enables cell repopulation by combining migration and proliferation. Data can be collected using a time-lapse microscope to record the results, or a micrograph can be used in defined conditions. To simulate actual wound sources, many wounding techniques are used in 2D wound healing assays, including scratching, stamping, thermal wounding, electrical wounding, optical wounding, and chemical wounding. The characteristics of each wounding experiment and the distinct benefits and drawbacks of various induction techniques are described in the following paragraphs.

Mechanical wounding using a scratching technique is applied on a confluent monolayer cell using a pipette tip, razor blade, 25-gauge needle in a standardised jig, 23-gauge stub adapter, rotating silicone tip, toothpick, cell scraper, or metallic microindenters by creating a linear artificial scratch to mimic the wound. The technique is simple and easy to apply. Many of the innovative methods available today are derivatives of this assay. However, the creation of artificial wounds using this method produces uneven wounded areas with accumulations of debris on the scratch edges (Martinotti and Ranzato, 2020; Kauanova et al., 2021). Scratching means the possible destruction of extracellular matrix coatings on the cell culture dish.

The second mechanical wounding method is stamping, which is the application of pressure on a defined area. In contrast to the scratch technique, stamping allows an artificial wound to be any shape, according to the mould. The cell culture matrix coating remains intact and the influence of cell debris on migration can be monitored. The major disadvantage of the stamping technique is the uneven manual pressure applied during the stamp (Stamm et al. 2016).

Furthermore, wounds on single systems can be created using the thermal wounding technique, which involves the application of a notable heated aluminium stamp. This technique is mostly applicable to wound burn and healing studies. However, the method produces uneven heat and mechanical transfer, as well as temperature deviation (Kokolakis et al., 2020). Differences in the heated stamp contours lead to low reproducibility. Hence, this thermal technique requires a control group (a stamp at room temperature) to determine mechanical damage.

Electrical wounding is another sensational wounding technique that involves the use of pulses of a recorded current of high voltage, which leads to electroporation (electric cell-substrate impedance sensing). In contrast to the previously described techniques, electrical wounding eliminates human error since the wound is created in automatic mode and allows real-time measurement. The destruction and regrowth of cells measured using impedance data lead to reproducible data. Yet the technique also has several disadvantages, including the difficulty of detaching/destroying the confluent cell layer, changes in adhesion and cell density that alter the impedance measurement, as well as heat development that potentially affects cell viability and low throughput (Polak et al., 2014; Semenov et al., 2016). Lastly, optical wounding can be used to create wound on a monolayer/2D wound healing assay using a laser beam. Like electrical wounding, this technique is reproducible across experiments because it has high throughput and is conducted in a sterile environment (Stamm et al., 2016). In addition, the need for electrical and laser beam applications requires specialised equipment to perform the wounding.

Angiogenesis, granulation tissue development, and reepithelialisation are just a few of the processes aided by cell migration in the wound healing process. Keratinocytes, fibroblasts, microvascular endothelial cells, melanocytes, and macrophages work together to regulate damage healing with several growth factors. Cells are connected as a network of scaffold and multicellular strands during the healing process, and cell migration must preserve intercellular connections and cell polarity. The migration starts a few hours after “cell damage”, where the epidermal cells become polarised. The actin cytoskeleton generates projections that resemble pseudopodium and are oriented towards the wound site’s open space. The scratch assay is created based on the migration phase, in which a confluent cell monolayer is mechanically scratched to introduce a wound. After administering medicinal substances, the healing process is observed, and information is collected based on the closure of artificial wounds, which are photographed under a microscope and analysed using image-analysis systems. This assay is frequently used in drug development laboratories because it is quick, practical, straightforward, and repeatable. The assay is commonly used to evaluate the migratory activities of plant extracts, natural compounds, or drugs to develop them for treating wounds (Martinotti and Ranzato, 2020; Kauanova et al., 2021).

In cancer development and progression, invasion and metastasis occur when tumour cells disseminate from a primary tumour, spreading through the circulatory and lymphatic systems. This makes cell migration an important step in cancer metastasis (Pijuan Marquilles et al., 2019). The scratch assay is an easy and simple way to study the migrations of cancer cells using different types of cell lines, such as carcinoma, sarcoma, leukaemia, lymphoma, myeloma, brain, and spinal cord cells. The scratch assay enables the observation of cancer cells that are about to migrate when a scratch is made in a cell monolayer, whether an anticancer chemical is present or not. Images of the fictitious gaps or scratches formed in cancer cells that have been treated with the potential anticancer treatment or control can be compared or assessed at regular intervals. A significant gap after the trial period, which showed the cancer cells’ ability to migrate, suggests a promising anticancer action. A time-lapse microscope and image analysis software monitor and record the migration course. Using gold particles, researchers have created the migration scratch assay (Leu et al., 2012) and the Green Fluorescence Protein (GFP) marker (Grada et al., 2017). Genes denoted using this marker can be followed to ascertain how a particular gene affects the movement of migrating cells. However, the scratch test experiment does not consider the chemical gradient affecting how cells migrate. Consequently, although ascertaining the migratory behaviour of keratinocytes takes longer, this test has not replaced the other widely used techniques to analyse cell migration, such as the Boyden Chamber chemotaxis experiment (Guy et al., 2017), endothelial (Michaelis, 2014), and fibroblasts (Cen et al., 2021).

Several Boyden Chamber products are commercially available. Cells are permitted to move to the lower compartment containing a chemoattractant in the two chambers of the device, which comprise two medium cases separated by a microporous membrane. After a specific time, cells move through the pores and into the lower chamber compartment. The number of cells carried through the pores is counted once the membrane has been fixed and stained (Chen, 2005).

### 3.2. Multicellular systems: cocultures and 3D wound healing assays

Three-dimensional cell cultures are made up of cells that have been joined and moulded into a 3D shape with the help of a surrounding medium or specialised conditions to maintain the shape. Three-dimensional cell cultures enable more cell-to-cell and cell-to-extracellular matrix interactions (Teimouri et al., 2018). Cell-cell interactions can be studied in a coculture system (Stamm et al., 2016). Cell cocultures in vitro tissue models are commonly used to explore how cells interact. These models depict a wide range of human body processes, including development, homeostasis, regeneration, and disease (Vis et al., 2020).

The techniques for producing 3D cell cultures are often classified into two categories: those that employ scaffolding and those that do not. The latter are thought to provide a better approximation of in vivo activity because the cells assemble on their own. In 3D cell cultures, when cells aggregate, they produce what is referred to as a spheroid. The hanging drop method, agitation-based approaches, and forced-floating method, as well as the utilisation of scaffolds, microfabricated 3D culture systems, and bioprinting technologies, are some of the methods that have been utilised in 3D cell cultures (Teimouri et al., 2018).

**Figure 3:**
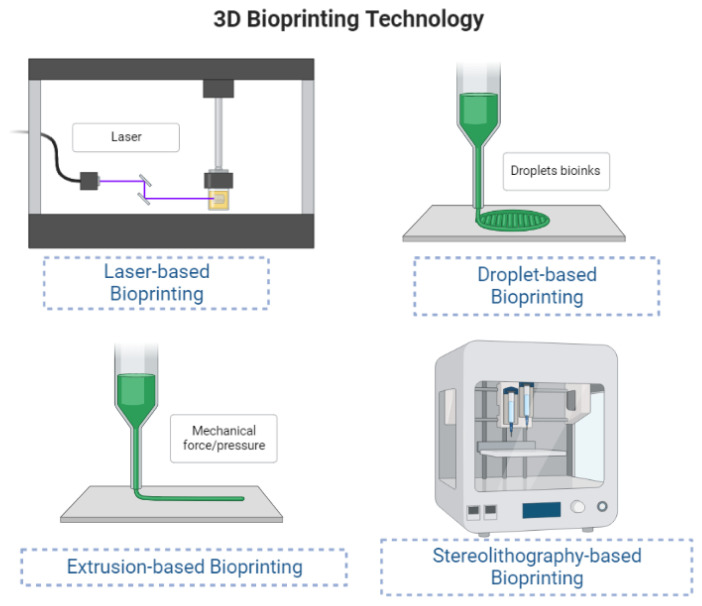
Summary of 3D-bioprinting technologies used for wound healing.

This section outlines the use of novel natural or synthetic biomaterials in 3D wound healing approaches and the design of cell-laden bioinks mixed with medicinal substances that have paved the way for effective wound therapy and management, including the engineering of skin substitutes and skin regeneration. The primary benefits of printing technologies include the combination of many bioactive chemicals and cells with polymers, the construction of complex scaffold designs, quicker healing times, and personalised wound dressings (Tabriz and Douroumis, 2022). Tissue 3D-bioprinting is an additive manufacturing process used to construct biocompatible 3D structures that resemble natural systems using a computer-generated design. Previous skin regeneration procedures are outperformed by 3D-bioprinted dermal replacements in terms of their automation and standardisation for clinical applications, as well as their accuracy due to the inclusion of living cells, growth factors, and other biomolecules. Figure 3 depicts the cellular bioprinting approaches that combine bioinks with live cells to create artificial scaffolds or tissue constructs. These processes can be divided into four types: laser-based, droplet-based, extrusion-based, and stereolithography-based bioprinting (Antezana et al., 2022).

Three-dimensional wound healing assays have been designed to ensure the greater physiological relevance of studies on topics such as morphology, signalling, migratory behaviour, and metabolic function, compared to two-dimensional wound healing assays. As a result, 3D wound healing tests can simulate normal wound physiology better than 2D wound healing assays.

The largest organ, the skin, is highly prone to injuries caused by severe burns and conditions like dermatitis or diabetes. Total-thickness wound patients are physically and financially burdened. Given that they trigger no allergic or harmful reactions, biodegradable patches have been used to treat wounds and are regarded as a feasible wound healing care/management.

A recent study used an extrusion-based printing technique to produce a 3D patch that mixes a gelatine-based hydrogel with a well-known natural antibacterial, Manuka honey. The patch demonstrated antimicrobial activity, the elevated proliferation of human dermal fibroblasts and human epidermal keratinocytes, as well as the promotion of angiogenesis (Brites et al., 2023).

Meanwhile, in another study, 3D-printed gelatine-alginate hydrogel dressings showed the best combination of mechanical properties, hydration activity, and in vitro biological reactivity. These dressings contained 75% gelatine and 25% alginate. Compared to a nonprinted hydrogel with the same composition and standard of care, the in vivo results using the most efficient dressing showed that the 3D-printed porous pattern had more positive effects on wound healing, including faster wound closure, regenerated hair follicles, and nontraumatic dressing removal (Fayyazbakhsh et al., 2022).

Furthermore, the latest research on 3D wound healing demonstrates the development of novel angiogenic 3D-bioprinted peptide patches and 3D-bioactive glass fibre scaffolds that have exhibited beneficial healing effects (Norris et al., 2020; Guan et al., 2022). Artificial skin has been developed for wound healing studies, such as Apligraf™, Novartis, East Hanover, NJ, Hyalograft 3D, and the TissueTech Autograft System. Some models are intended for dermatological testing, such as EpiDermFT™, Phenion^®^ FT Model, and StrataTest^®^ (Groeber-Becker et al., 2012).

### 3.3. Organ culture

Several substitute technologies are becoming accessible as skin safety investigations shift away from conventional animal-based methods. One such technique uses human skin as an organ. Organ culture is a simple and reasonably priced method for preclinical safety evaluation. Organ culture should be used when the list of drugs to be assessed is minimal and when simpler models have narrowed the dose range, even if it is unlikely to replace high-throughput enzyme tests or monolayer culture equivalent skin cultures for the first compound assessment. Organ-cultured skin also offers avenues for mechanistic research (Varani, 2012).

Organ culture offers numerous advantages for wound healing studies, including the preservation of the three-dimensional structure and cellular composition of the tissue or organ, as well as precise control over various experimental conditions such as temperature, humidity, nutrient supply, and exposure to specific stimuli. Moreover, using organ culture enables highly reproducible experiments to be conducted using standardised protocols in identical experimental conditions, complemented with sufficient flexibility and versatility in terms of the use of different tissue types (skin, cornea, or blood vessels). Organ culture is more cost-effective and reduces the strong reliance on animal models for wound healing studies, thus eliminating the need for the complex ethical considerations pertaining to live animal experimentation. Organ culture also allows an extended experimental duration, which is beneficial for assessing various stages of chronic wound healing (inflammation, proliferation, and remodelling). Using organ culture enabled manipulation and intervention studies to be conducted, allowing researchers to introduce specific compounds, drugs, growth factors, or genetic modifications to the tissue or organ culture system during wound healing studies at molecular levels. These advantages have contributed to a deeper understanding of wound healing mechanisms and facilitated the development of novel therapeutic approaches. Organ culture models have been used to evaluate promising wound healing therapies, including dermal substitutes (Van Kilsdonk et al., 2013), antiinflammatory interleukin 10 (Balaji et al,. 2014), inflammatory interleukin 27 (Yang et al., 2017), human mesenchymal stem cell secretome (Carter et al., 2019), a heparin-binding EGF-like growth factor (Thönes et al., 2019), and the cytostatic agent Mitomycin C (Schumann et al., 2021). Significant advancements in scarless wound healing have been made in the last decade due to the adoption of new ideas from mechanobiology and immunology. Mechanical stress signals and immune responses clearly play crucial roles in determining the wound healing mode from the complete integumentary organ system (IOS) to the regeneration and scarless wound healing mechanism, which only occurs in particular species, body sites, and developmental stages. The development of innovative human skin analogues and organoids that mimic cell-cell interactions and tissue-scale tensional homeostasis has enabled us to assess the morphology, functioning, medication response, and wound healing of skin tissue (Kimura and Tsuji, 2021).

## 4. In vivo models

In vivo or animal models provide essential information about wound healing by exploring its cellular and molecular pathways. Furthermore, these models are the most predictive for assessing the efficacy and safety of various therapeutic drugs/agents, and they serve as appropriate alternatives for wound healing evaluation. Assessment using an in vivo model entails creating wounds in laboratory animals and then observing wound closure and healing over time. A wide range of animal models (such as rats, mice, rabbits, pigs, and zebrafish) have been exploited during in vivo wound healing evaluation in which wound dressings were employed. However, the differences in the anatomical and physiological functioning of animals and humans mean there is no unanimity on the choice of a single animal model. When using animal models, researchers should adhere to the 3R (replacement, reduction, and refining) principles to ensure the ethical and compassionate treatment of the animals. Wound healing efficiency is generally influenced by the type of wound dressing, the animal model, the wound location, and the microbiota (Elliot et al., 2018; Ahmad, 2023).

Studies published from 1993 to 1997 were compared to publications dating from 2013 and 2017, revealing significant variations in model and species usage. More of the later experiments were using mouse and human models, while the use of pig models had remained steady. Experiments using rabbit and rat models had decreased in the most recent time period studied, compared to the previous two decades (Parnell and Volk, 2019).

By enabling the process of angiogenesis in skin wound healing to be studied, in vivo models recapitulate wound healing, allowing further distinctions between potential direct studies, such as intravital fluorescence microscopy (IVM), and indirect methods (histological or biomechanical analyses of tissue samples from an in vivo skin wound model). While the dorsal skin fold chamber of a mouse or hamster model is frequently employed directly to visualise healing processes, tissue samples from any of the models described can be used in indirect approaches (Grambow et al., 2021).

### 4.1. Types of animal wound models

Various animal models can be utilised for wound healing investigations, including pigs, rats, mice, or rabbits, depending on the study goal, as each species has a unique immune system function, tissue shape, and physiology. A thorough selection of acceptable animal models is required to ensure that the use of real animals is justified. Simultaneously, the generated model must imitate human wounds with relevant illness conditions to depict clinical circumstances. Other aspects to consider are the cost, competence and convenience of handling, sufficiency of biopsy samples, animal husbandry, ethics application, and study duration. Porcine models are appropriate animal models for wound healing (Ahmad, 2023).

### 4.2. Wound healing assessment techniques

The most popular techniques for assessing wound healing in vivo wound models that employ wound dressings include visual examination for measuring changes in wound size; wound healing rate analysis using epithelialisation, vascularisation, and ECM deposition; biochemical assays (collagen metabolism, oxidative stress, or myeloperoxidase); and histological and immunohistochemical studies (the release of cytokines and growth factors) (Masson-Meyers et al., 2020).

These assessment techniques give information about the properties of the wound bed, tissue growth, degree of scarring, vascularisation, and pathological diseases. Based on the approach, wound healing evaluation methods are divided into noninvasive and invasive. The former includes visual macroscopic observation, which provides imaging for wound analysis (regular photography, image analysis software); the wound healing rate (change in wound surface area and wound tracing method); and biophysical wound assessment utilising in vivo imaging techniques like optical coherence tomography (OCT), diffuse near-infrared spectroscopy, and confocal laser scanning microscopy (Yazdanpanah et al., 2021).

In contrast, invasive wound evaluation techniques include biochemical, histological, and immunological approaches. Myeloperoxidase assays (for evaluation of the inflammatory phase and neutrophil recruitment/accumulation), the oxidative stress profile (reactive oxygen and nitrogen quantification), and N-acetylglucosaminidase are a few examples of the biochemical assays used to measure different macromolecules (macrophages assessment) (Barreto et al., 2016; Chiang et al., 2017).

Meanwhile, haematoxylin and eosin staining is the most frequently used histological technique for qualitatively assessing the pathological conditions and process of wound healing (Liu et al. 2019). The immunological techniques include immunohistochemistry examinations (different staining methods utilising monoclonal antibodies for collagen localisation and reepithelialisation assessment) and ELISA assays (determination of various inflammatory mediators, growth factors, and cytokines) (Sun et al., 2022). Flow cytometry and macrophage polarisation studies are also used to better understand the cellular processes during wound healing. These invasive and non-invasive approaches provide comprehensive information about wound healing progression (Ahmad, 2023).

## 5. Conclusion

Numerous in vitro research studies can be chosen from those conducted in various laboratories. The decision on which assay to use will be based on the project objectives, which might bridge a knowledge gap about the project challenges. When deciding on an acceptable assay method, it is advisable to assess the relevance of each assay to the study problem. For instance, while investigating drug discovery, it is preferable to use a quick screening assay that uses monolayer cells rather than develop a 3D assay multicellular cell. Moreover, it is prudent to examine whichever stage of the healing process is being addressed and how much evidence is required to corroborate the observations. Equally significant is the expense of carrying out project activities. It is also critical to evaluate the actual healing process being researched, whether the wound is due to acute damage, blunt damage, or a burn. Considering these characteristics should enable the appropriate model to be selected, which would be an essential aid in the study of wound healing promotion.

### Future perspectives

A multitude of parameters in the optimum wound healing model must be considered. Although it has been stated that no wound healing model is superior to others, the complications of a wound environment mean that animal models will always be preferable to in vitro models. Rodent animal models will continue to be the primary model for wound healing research since they are inexpensive, easy to handle, and dependable. Mice, rabbits, zebrafish, and pigs are the ideal animal models for pharmacological testing before moving on to human clinical trials (Ribitsch et al., 2020).

Finding molecular target genes that can be enhanced to accelerate the body’s natural healing process is the primary goal of the current research on wound healing. A complete approach prioritising proper dressing and local care, nutritional support, and hyperbaric oxygen therapy in severe cases is necessary to promote effective wound healing in the most challenging situations in this field (Ayavoo et al., 2021). In addition, 3D-bioprinting technology has various advantages when creating a human skin-equivalent wound model. This model is considered the best prospective platform for pharmacological tests and dermatological research that may improve throughput and scalability in manufacturing equally damaged skin samples (Jara et al., 2021).
